# Ultrathin SmCo_5_ nanoflakes with high-coercivity prepared by solid particle (NaCl) and surfactant co-assisted ball milling

**DOI:** 10.1038/srep25805

**Published:** 2016-05-13

**Authors:** Wen-Liang Zuo, Xin Zhao, Tong-Yun Zhao, Feng-Xia Hu, Ji-Rong Sun, Bao-Gen Shen

**Affiliations:** 1 State Key Laboratory of Magnetism, Institute of Physics, Chinese Academy of Sciences, Beijing, 100190, People’s Republic of China

## Abstract

The ultrathin SmCo_5_ nanoflakes with average thickness smaller than 50 nm are prepared by a novel method of solid particle (NaCl) and surfactant co-assisted ball milling. The as-prepared nanoflakes exhibit a narrower thickness distribution of 10–50 nm and high coercivity of 23 kOe. The possible formation mechanism of nanoflakes are proposed. Temperature dependence of demagnetization curves indicate that the magnetization reversal may be controlled by both nucleation and pinning. The results of X-ray powder diffraction and magnetic measurement for aligned SmCo_5_ nanoflakes resin composite indicate that the nanoflakes have a high texture degree. The ultrathin thickness and high coercivity are beneficial for preparing the high performance soft/hard coupling magnets and nanocomposite magnets.

Nanostructured Co-based rare-earth permanent materials with high coercivity and strong texture have drawn much attention due to their high temperature application and which can be used for preparing the high-performance soft/hard exchange coupled permanent magnet and high-density data storage media^1^,^2^,^3^,^4^. Furthermore, the particle sizes are the smaller the better as long as they are larger than the supperparamagnetic transition size for soft/hard coupling magnet or nanocomposite magnet^5^. However, synthesis of high-quality rare-earth compounds with small size turns out to be a non-trivial task due to the great chemical instability^5^,^6^. Wet-chemical methods are commonly used for preparing the nanoparticles with controlled size. However, until now it is still limited success in obtaining high coercivity and high purity samples^4^,^5^. Physical method of cluster-deposition is also used to produce the textured rare-earth compounds nanomagnets^7^. Nevertheless, not only the equipment is expensive, but also the yield is low. Surfactant-assisted ball milling (SABM) is also used for preparing the rare-earth based hard magnetic nanomaterials^6^,^8^. Unfortunately, most of the experiment results exhibit the large thickness and wide size distribution^8^,^9^,^10^, although the further centrifugal separation can be used obtain smaller size^6^, both the low output and low coercivity limit its development. In recent work, we prepared the SmCo_5_ nanoflakes with ultrahigh coercivity of 26.2 kOe using a multistep SABM^11^. However, the thickness of that nanoflakes is still about 50–200 nm, the further decrease in thickness can cause the speedy decrease of coercivity. In this articles, a novel method of the solid particle (NaCl) and surfactant co-assisted ball milling (SPSABM) are used for preparing the ultrathin rare-earth permanent magnetic (SmCo_5_) nanoflakes with narrow size distribution and high coercivity. The results indicate that it is a fine method for decreasing the thickness of nanoflakes but still keep the high coercivity.

## Results and Discussions

**Figure 1:**
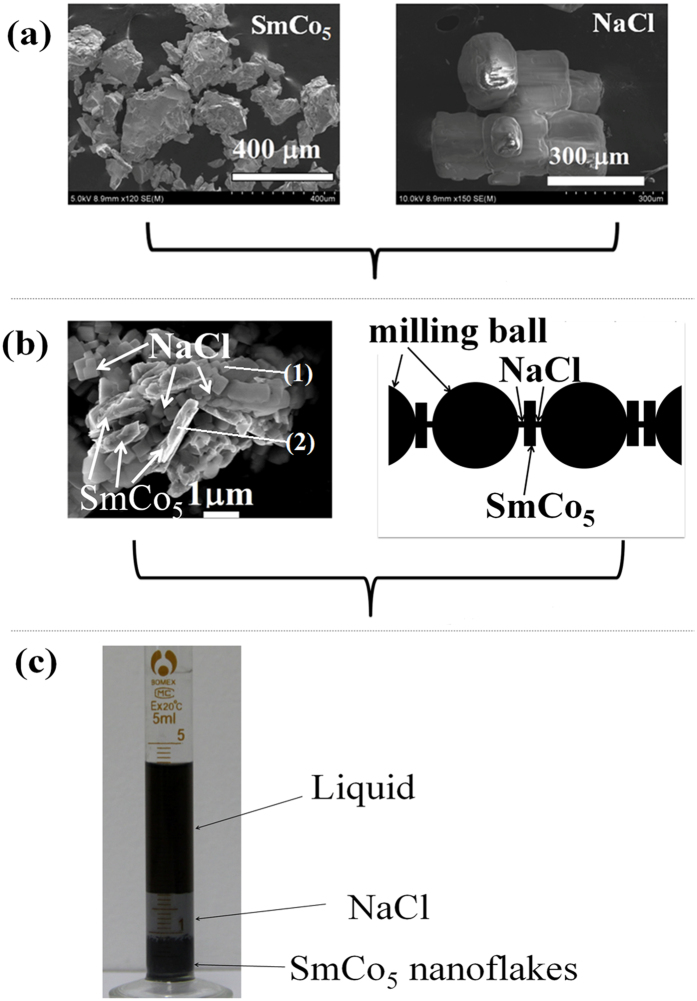
(**a**) the start powders of SmCo_5_ compound and NaCl, (**b**) the SEM images of ball milled produces with 8 h ball milling (left), the marked numbers in image are the positions of EDS analysis, and the corresponding schematic plot (right), where square, rectangle and rectangle represent the solid particle (NaCl), milling materials (SmCo_5_ nanoflakes) and milling ball, respectively, (**c**) the photo of milled produces in test-tube after standing for 4 h.

Figure 1 shows the evolution mechanism of SmCo_5_ nanoflakes with the SPSABM time from 0 to 20 h. The start powders of SmCo_5_ compound and NaCl are shown in Fig. 1(a). It can be seen that the SmCo_5_ compound shows irregular shape with the size of 50–400 μm, while the NaCl exhibits regular cube with size about 150 μm. With the increase of milling time reaches 8 h (See Fig. 1(b)), the solid particle (NaCl) and the milling materials (SmCo_5_) are grated into smaller size. The elemental analysis of energy dispersive spectroscopy (EDS) is shown in Table 1. It is interested that NaCl particles become smaller than SmCo_5_ flakes. In this conditions, the NaCl nanoparticles will easily fill in the interspaces of SmCo_5_ flakes and milling balls, the according schematic is shown in the right of Fig. 1(b), where square, rectangle and semicircle represent the solid particle (NaCl), nanoflakes (SmCo_5_) and milling ball, respectively. It is obvious that the NaCl nanoparticles will be used as a pivot and the intensity of pressure in the pivot of nanoflakes will dramatically increase, which promotes the nanoflakes further smashing but has little effect on other part of the nanoflakes, it also means the breaking effect may be only at the point rather than the whole plane (smaller contact area), which can decrease the percentage of crystal structure defects of the nanoflakes than the traditional ball milling at the same condition. It should be noted that the schematic only shows three types of possible situation, and realistic situation may be more complex. When the milling time reaches to 20 h, the ball milling product is collected in the test-tube and let it stand for 4 h, the picture is shown in Fig. 1(c). It can be seen that the milling product exhibits obvious layering. In the bottom of cylinder (black regions) is mainly SmCo_5_ nanoflake, the middle (Grey white regions) is mainly NaCl, and the top (black regions) is the liquid (include Heptane, Oleylamine, oleic acid, and very small amount of SmCo_5_ and NaCl nanoparticles). The spontaneous layering is very important for further purification and could be due to the density of NaCl smaller than that of SmCo_5_. The bottom slurry of test-tube is washed three times using Heptane, then collected for measurement.

**Figure 2:**
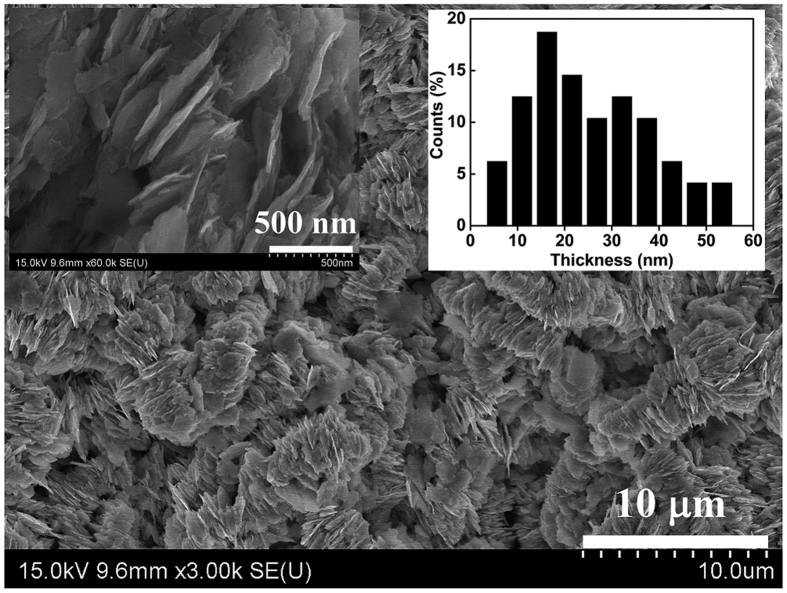
The SEM image of SmCo_5_ nanoflakes milled for 20 h, the top left and top right are the enlarged image and histogram for selected area, respectively.

**Figure 3:**
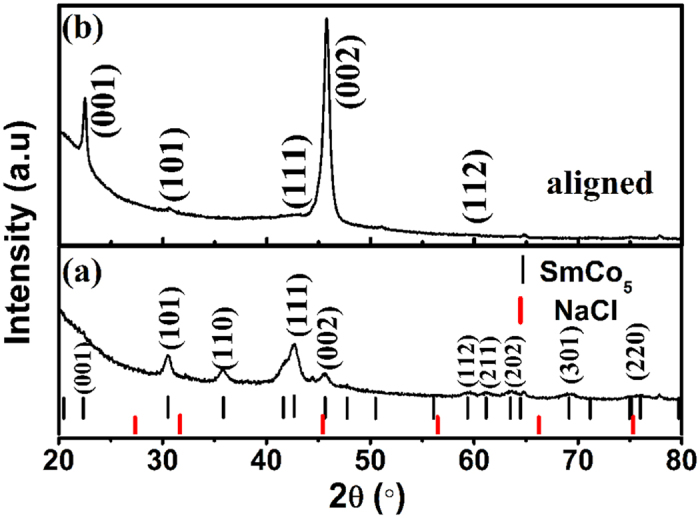
The XRD patterns of (**a**) randomly oriented SmCo_5_ nanoflakes, (**b**) aligned SmCo_5_ nanoflakes resin composite.

The SEM image of the as-milled nanoflakes is shown in Fig. 2, the top left and top right are the enlarged image and histogram of selected area, respectively. It can be seen that the thickness of nanoflakes are mainly distributed in the range of 10–50 nm, which indicates that the SPSABM are advantageous for obtaining the narrower size distribution and smaller thickness than the traditional SABM^9^,^10^,^11^,^12^,^13^. Both the narrower size distribution and smaller thickness are favorable for preparing the high performance soft/hard coupling magnets and nanocomposite magnets. Furthermore, there is no trace of NaCl in the nanoflakes, which will be demonstrated by XRD (See Fig. 3(a)) and is important for actual production and application. Moreover, the nanoflakes show smaller diameter (1–2 μm) compared with the usual SABM with high energy^8^,^9^,^10^, which is beneficial for further increasing the coercivity due to decreasing the effect of local demagnetization fields. As usual, the nanoflakes also form “kebab-like” morphology due to the *c*-axis texture and magnetostatic interaction, which implies that the easy magnetization direction of as-milled SmCo_5_ nanoflakes is perpendicular to the surface of the flakes. The XRD pattern of as-milled SmCo_5_ nanoflakes with randomly oriented is shown in Fig. 3(a). It can be seen that the as-milled powder mainly exhibits the hexagonal SmCo_5_ phase (JCPDS PDF#65-4844) and no the trace of NaCl (JCPDS PDF#05-0628). It agrees with the result of SEM (See Fig. 2) and indicates that the NaCl and SmCo_5_ can be easily separated. In addition, the XRD pattern of aligned sample is also shown in Fig. 3(b). It can be seen that the diffraction intensity of (00*l*) crystalline planes remarkably enhances while the other peaks almost disappear, suggesting that the aligned sample possesses a strong (00*l*) alignment (the easy magnetization directions along the *c*-axis). The normalized *c*-axis alignment degree is 99% for the aligned SoCo_5_ nanoflakes resin composite according to the papers^14^,^15^,^16^, the lattice parameters (PDF#65-4844) and intensity of diffraction peaks are obtained from Fig. 3. Which indicates that the ultrathin SmCo_5_ nanoflakes maintain a high texture degree.

**Figure 4:**
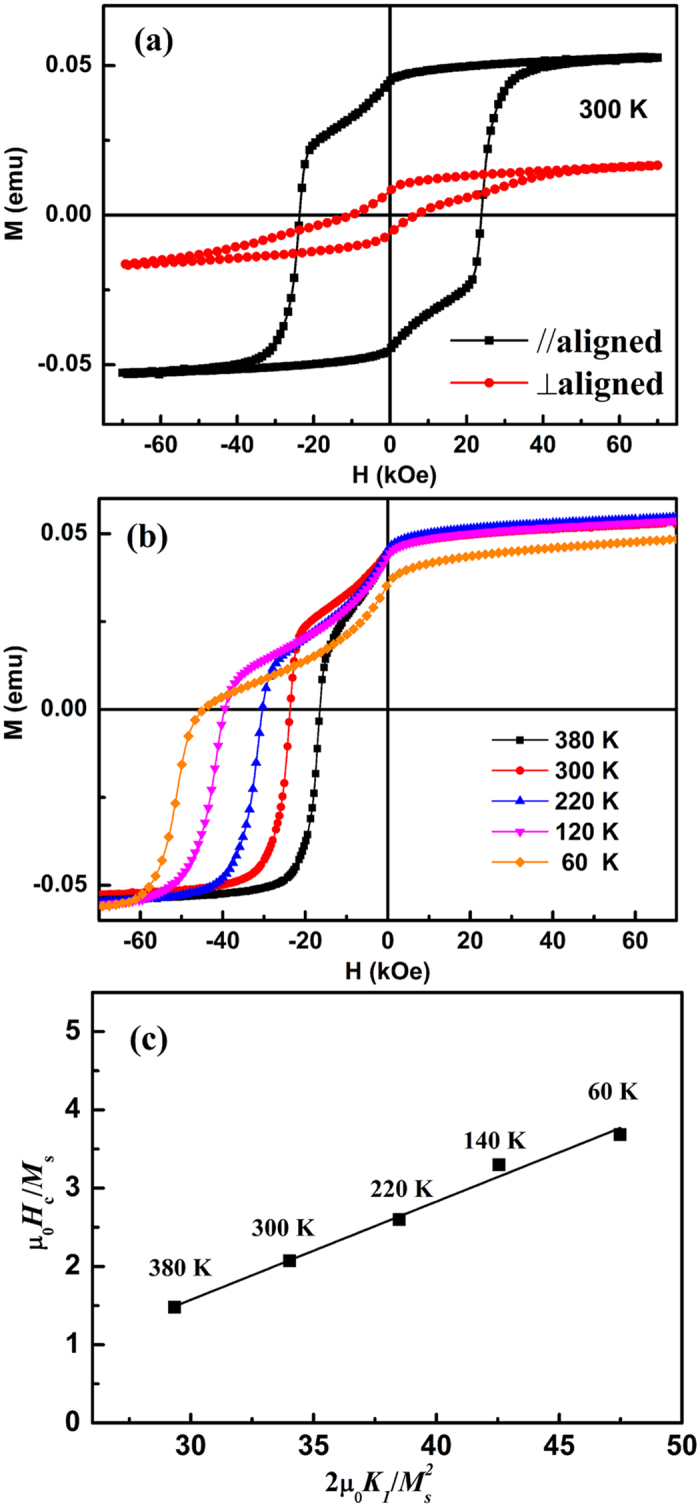
(**a**) The hysteresis loop, (**b**) temperature dependence of demagnetization curves and (**c**) *μ*_0_*H*_*c*_/*M*_s_ against 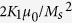
on different temperature for aligned SmCo_5_ nanoflakes resin composite.

Figure 4(a) shows the hysteresis loops of aligned SmCo_5_ nanoflakes resin composite. Firstly, the obviously anisotropy magnetic behaviors are observed, which indicates that the nanoflakes have a large magnetic anisotropy. Secondly, a high coercivity of 23 kOe is obtained along the easy axies, which is about 40% higher than the traditional SABM^8^, and gives the nanoflakes great potential application. In addition, we also calculate the average misalignment angle, *φ* = arctan[2*M*_r_(⊥)/2*M*_r_(||)]^17^,^18^, where *M*_r_(⊥) and *M*_r_(||) are the remanence of perpendicular and parallel direction of the easy axis, respectively. The misalignment angle φ = 19.6°, which indicates that the nanoflakes with SPSABM have a high texture degree compared with the experiment results of SABM in the magnetic field^17^,^19^. In here, it should be known that the calculated results of texture degree form XRD and VSM are consistent although the values are seemingly different. Which are mainly due to two different evaluation system, the data from XRD shows the alignment of crystal texture, while the data from M-H loops reflect the alignment of magnetization (or the magnetic domain). As the fact that there are slight nanopaticles or nanoflakes with very small size in the grain boundaries^8^,^19^, that incline to random orientation due to the amorphous and superparamagnetic, and they couldn’t be checked by XRD but is very sensitive to reverse magnetization process (namely, the domain structure), especially for the hard magnetic materials. In addition, the effect of amorphous and superparamagnetic can also be reflected in the demagnetization curves. The approximately linear decrease of magnetization in the second quadrant indicates that there really exist some low coercivity materials together with the high coercivity nanoflakes, which most possibly are the amorphous SmCo_5_ materials according to the ball milling method and other paper^8^,^13^. However, the phenomenon is more inconspicuous than that of the high energy SABM^8^,^19^, which indicates the SPSABM could be beneficial for decrease the defect and obtaining the ultrathin SmCo_5_ nanoflakes with high coercivity. In order to study the mechanism of high coercivity, the temperature dependence of demagnetization curves are shown in Fig. 4(b). According to the micro magnetic model, the coercivity can be generally expressed as^20^,^21^: *H*_*c*_ = *α*_*K*_*α*_φ_2*K*_1_/*μ*_0_*M*_*s*_ − *N*_*eff*_*M*_*s*_, where *K*_1_, *N*_*eff*_, and *M*_s_ are the first-order anisotropy constant, the effective local demagnetization factor and the saturation magnetization, respectively. The coefficient α_K_ represents the effect of the sample microstructure, especially for the inhomogeneous intrinsic material parameters, and *α*_φ_ describes the information of the easy axis misaligned. These parameters can be determined by linear fitting *μ*_0_*H*_*c*_/*M*_*s*_ against 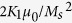 (See Fig. 4(b)). The temperature dependent values of *K*_1_ and *M*_s_ are from the SmCo_5_ single crystal^22^. The obtained α_K_*α*_φ_ and *N*_*eff*_ are 0.125 and 2.19, respectively. The value of 0.125 is mainly attributed to the microstructure parameter α_K_ due to the small misalignment angle for the aligned samples, and this value is similar (and slightly smaller) to the multistep ball milled SmCo_5_ nanoflakes, which could be attributed to the slightly smaller misalignment angle^11^. And this also indicates that the ultrathin SmCo_5_ nanoflakes have a similar microstructure compared with the multistep ball milled SmCo_5_ nanoflakes. In addition, the α_K_*α*_φ_ value of 0.125 indicates that the magnetization reversal may be controlled by both nucleation and pinning (α_K_ < 0.3)^20^,^23^. The value of *N*_*eff*_ is larger than 1 and slightly smaller than that of the multistep ball milled SmCo_5_ nanoflakes^11^, which could be due to the smaller thickness^24^. Meanwhile which also indicates that the smaller stray field is existed in the ultrathin SmCo_5_ nanoflakes resin composite compared with that of multistep ball milled results due to the larger local demagnetization effect (larger aspect-ratio)^19^,^20^,^23^.

The ultrathin SmCo_5_ nanoflakes are prepared by a novel method of solid particle (NaCl) and surfactant co-assisted ball milling. The as-prepared nanoflakes exhibit a narrower thickness distribution of 10–50 nm and high coercivity of 23 kOe. The result of XRD and VSM for aligned SmCo_5_ nanoflakes resin composite indicates that the ultrathin nanoflakes have a high texture degree. The small thickness, narrow size distribution and large coercivity are beneficial for preparing the high performance soft/hard coupling magnets and nanocomposite magnets. The low cost of equipment and high output further give the nanoflakes greatly potential practical application.

## Methods

SmCo_5_ ingots were purchased from Taiyuan Tianhe Hi Tech Co Ltd, and were annealed at 1173 K for a week under vacuum, then ground down to less than 400 μm as the starting powders. The SPSABM experiment was performed using a GN-2 BM equipment with the speed was about 250 rpm. Oleylamine (80–90%) and oleic acid (99%) were used as surfactants, the total amount was 20% to the weight of the starting powders (Oleylamine and oleic acid was 1:1). NaCl was used as the solid particle, the size was about 150 μm, and the weight was 3:1 compared with the starting powders. Heptane (99%) was used as the carrier liquid. The aligned SmCo_5_ nanoflakes resin composite was prepared by mixing the as-milled nanoflakes with epoxy resin, and placing them into a 20 kOe magnetic field until the epoxy resin solidifies. The phase structure was examined by the X-ray powder diffraction (XRD) with Cu Kα radiation at room temperature. Morphology was analyzed by scanning electron microscope (SEM). Magnetic properties were measured by a SQUID VSM with the maximum field of 70 kOe.

## Additional Information

**How to cite this article**: Zuo, W.-L. *et al.* Ultrathin SmCo_5_ nanoflakes with high-coercivity prepared by solid particle (NaCl) and surfactant co-assisted ball milling. *Sci. Rep.* **6**, 25805; doi: 10.1038/srep25805 (2016).

## Figures and Tables

**Table 1:** Elemental analysis of EDS for the marked positions in left of Fig. 1(b).

Position	Sm (at.%)	Co (at.%)	Na (at.%)	Cl (at.%)
(1)	0	0	52.01	47.99
(2)	17.73	82.28	0	0
